# Exploiting the inter-strain divergence of *Fusarium oxysporum *for microbial bioprocessing of lignocellulose to bioethanol

**DOI:** 10.1186/2191-0855-2-16

**Published:** 2012-03-15

**Authors:** Shahin S Ali, Mojibur Khan, Brian Fagan, Ewen Mullins, Fiona M Doohan

**Affiliations:** 1 Molecular Plant-Microbe Interactions Laboratory, School of Biology and Environmental Science, University College Dublin, Dublin 4, Ireland; 2 Dept. of Crop Science, Teagasc Crop Research Centre, Oak Park, Carlow, Ireland; 3 Institute of Advanced Study in Science and Technology, Guwahati, India

**Keywords:** Fungal biomass, Endoglucanase, Endoxylanase, Consolidated bioprocessing, Solid-state cultivation

## Abstract

Microbial bioprocessing of lignocellulose to bioethanol still poses challenges in terms of substrate catabolism. A targeted evolution-based study was undertaken to determine if inter-strain microbial variability could be exploited for bioprocessing of lignocellulose to bioethanol. The microorganism studied was *Fusarium oxysporum *because of its capacity to both saccharify and ferment lignocellulose. Strains of *F. oxysporum *were isolated and assessed for their genetic variability. Using optimised solid-state straw culture conditions, experiments were conducted that compared fungal strains in terms of their growth, enzyme activities (cellulases, xylanase and alcohol dehydrogenase) and yield of bioethanol and the undesirable by-products acetic acid and xylitol. Significant inter-strain divergence was recorded in regards to the capacity of studied *F. oxysporum *strains to produce alcohol from untreated straw. No correlation was observed between bioethanol synthesis and either the biomass production or microbial enzyme activity. A strong correlation was observed between both acetic acid and xylitol production and bioethanol yield. The level of diversity recorded in the alcohol production capacity among closely-related microorganism means that a targeted screening of populations of selected microbial species could greatly improve bioprocessing yields, in terms of providing both new host strains and candidate genes for the bioethanol industry.

## Introduction

Although much progress has been made in developing and optimising the many areas of biomass biorefining (Tian et al. 2010), the efficient production of bioethanol fuel from lignocellulosic biomass remains an obstinate challenge. Conventionally, it involves the thermo-chemical hydrolysis of hemicellulose, followed by enzymatic hydrolysis of cellulose and yeast-based fermentation of the resulting sugars. Alternatively, some microbes can enzymatically hydrolyse cellulose and hemicellulose to sugars and then ferment the released hexose and pentose sugars (glucose, mannose, galactose, xylose and arabinose), in a process called consolidated bioprocessing (CBP) (Lynd et al. 2005). The bottleneck with regard to CBP is the scarcity of suitable microorganisms that exhibit high-end efficiency with regard to both substrate utilisation and product formation. Much research is focussed on finding and designing such organisms, driven by the significant reductions in both capital and operational cost that they will bring to lignocellulose bioprocessing. In parallel, finding a way to reduce by-product accumulation such as xylitol, acetaldehyde, glycerol, formic, lactic, and acetic acids is also a major focus area of lignocellulosic bioethanol research.

Plant-pathogenic microbes have evolved the capacity to break down the lignocellulose present in host cell walls and thus breach the barriers to invasion and host colonization. The plant-pathogenic fungi *Fusarium oxysporum *and *Neurospora crassa *have been shown to facilitate the CBP of lignocellulose to bioethanol (Deshpande et al. 1986; Christakopoulos et al. 1991a; Dogaris et al. 2009). The species *F. oxysporum *represents a group of pathogenically and genetically diverse strains (Khilare et al. 2010). Singh & Kumar (1991) have described many distinguishing features of *F. oxysporum *in comparison to other organisms when it comes to fermentation of lignocellulosic materials to ethanol which makes it a preferred choice for further scientific studies. Christakopoulos et al. (1989) showed that three strains of *F. oxysporum *differed in their cellulase activities and went on to demonstrate that while this fungus can facilitate CBP, the conversion rate of cellulose to sugar is slow and is accompanied by a build-up of the undesirable by-product acetic acid.

The goal of this study was to determine if there was significant inter-strain variation in the ability of *F. oxysporum *to release bioethanol from lignocellulose medium, where the primary nutrient source was unprocessed wheat straw and bran (10:1 ratio). A population of 17 strains of *F. oxysporum *were isolated from agricultural soil, peat and plant samples collected from different regions in Ireland. These were confirmed as being genetically distinct based on the internal transcribed spacer (ITS) region of nuclear ribosomal DNA and elongation factor (EF)-1α gene. A simple solid-state culture technique was optimised for bioethanol yield and was used to assess inter-strain divergence in bioethanol and to determine if this trait correlated with either fungal: growth, enzyme activities or the formation of acetic acid and xylitol.

## Materials and methods

### Origin and maintenance of fungi

*F. oxysporum *strains were isolated from agricultural soil, peat and plant samples collected from different sites in Ireland between October 2007 to February 2008 (Additiona file 1: Table S1) using Komada's selective medium (Komada 1975) (see Additiona file 1 information for more details). Prior to use, fungal isolates were sub-cultured onto PDA plates and incubated at 25°C for 5 days.

### DNA extraction, F. oxysporum-specific PCR analysis and DNA sequencing

Genomic DNA was isolated using the rapid mini-preparation procedure described by Edel et al. (Edel et al. 2000). *F. oxysporum*-specific polymerase chain reaction (PCR) analysis was conducted using the primer pair PFO2/PFO3 and the conditions described by Edel et al. (2000) in order to amplify a 70 bp DNA fragment of the 28S rDNA D2 domain. PCR products (10 μl) were electrophoresed through 1% (wv^-1^) agarose gels containing 0.5 μgml^−1 ^ethidium bromide. Products were visualised using Imagemaster VDS and Liscap software (Pharmacia Biotech, San Francisco, CA).

Fragments of both the ITS region of nuclear ribosomal DNA (nrDNA) and EF-1α gene amplified using primer pairs ITS4/ITS5 and EF-1/EF-2 and the conditions described by White et al. (1990) and O'Donnell et al. (1998), respectively. PCR products were electrophoresed through 1% (wv^-1^) agarose gels containing 0.5 μgml^−1 ^ethidium bromide; bands were visualised by UV transillumination and were excised. PCR products were cleaned up using the mi-Gel Extraction Kit (Metabion, Germany) and were sequenced by Macrogen (Seoul, Korea). Consensus sequences (derived from forward and reverse sequences) were subjected to BLAST analysis (http://www.ncbi.nlm.nih.gov) (Altschul et al. 1997). Sequences were aligned using European Bioinformatics Institutes's ClustalW2 tool (http://www.ebi.ac.uk) (Larkin et al. 2007) and phylogenetic trees were generated using the Neighbor-joining method (Saitou & Nei 1987).

### Solid-state cultivation (SSC) on straw/bran

The carbon substrate used for most SSC experiments was based on non-alkali-treated wheat straw blended with wheat bran. Dry wheat straw (cultivar Einstein) was ground in a coffee grinder (Model 203C, KRUPS, Poland, Mexico), passed through a sieve (2 mm pore size) and blended with unprocessed wheat bran (particle size ≤ 3 mm) (10:1 ratio of straw to bran). The straw composition of cultivar Einstein was determined as follows: cellulose 38.46%, hemicellulose 27.50% and lignin 13.18% (Ali et al. unpubl data). One gram of the straw/bran blend was mixed with 5 ml minimal medium (see below; excluding a C-source) and autoclaved (121°C for 15 min) in a 100 ml Erlenmeyer flask. Except where otherwise stated below, the cultures were grown in a minimal medium earlier described by Mishra et al. (1984) (pH 5); they contained 91% initial moisture (vw^-1^) and were maintained at 25°C. Fungal conidial inoculums was produced in Mung bean broth as described by Brennan et al. (2005) and were re-suspended in the minimal medium at a concentration of 10^6 ^ml^-1^. Flasks were supplemented with either 4 ml of conidial suspension or minimal medium (negative controls). For the aerobic growth periods, Erlenmeyer flasks were plugged with non-absorbent cotton and covered with aluminium foil. For the oxygen-limited growth period, Erlenmeyer flasks were plugged with cork and sealed with parafilm.

SSC experiments were conducted in order to determine the influence of the following factors on the bioethanol production capacity of *F. oxysporum*: (a) the duration of SSC - the length of aerobic/oxygen-limited growth phases were varied as follows: 2/4, 3/4, 3/7, 4/2, 4/4 and 4/7 days; (b) minimal medium composition - SSC was conducted using the fungal media and associated pH earlier described by Uchida et al. (2003), Crawford (1987), Christakopoulos et al. (1991b), Mishra et al. (1984), Bollok and Reczey (2000) and Panagiotou et al. (2003); (c) temperature - SSC cultures were incubated at either 20, 25, 30, 32 or 35°C; (d) pH - SSC cultures were adjusted to initial pH 4, 5, 6, 7 or 8 and cultures were incubated at 35°C; (e) moisture content - adjusted to either 80, 85 and 91% initial moisture (vw^-1^) by adjusting the amount of minimal media added to the flask before autoclaving and cultures were incubated at 35°C. For each treatment, three replicate flasks were used and each experiment was conducted twice.

Shake flask cultivation of delignified straw were also conducted in order to determine the efficacy of *F. oxysporum *in producing ethanol from it. The culture conditions were not as above; those described by Christakopoulos et al. (1991a) were used in order to compare yields with those previously reported for other strains of *F. oxysporum*. In brief, fungal inoculum was grown aerobically in a 250 ml Büchner flask containing 50 ml minimal medium and 1% (wv^-1^) alkali treated cellulose and 0.15% (wv^-1^) wheat bran as carbon sources. After 3 days, cultures were amended with 1.5 g of dry-sterilised alkali-treated wheat straw and grown under oxygen-limiting condition for 4-6 days. For switching from aerobic to oxygen-limiting condition inside the flask, the cotton plugs were replaced with a rubber bung and sealed with paraflim while the hose barb was fitted with a silicon tube and the end was dipped in water filled beaker.

### Estimation of bioethanol

Following SSC, flasks were incubated at 4°C for 1 h in order to condense any synthesised alcohol. Then flasks were supplemented with 10 ml of sterile cold water, plugged with cork and incubated at 150 rpm, 25°C for 1 h. All ethanol extraction procedures thereafter were conducted in a cold room (4°C). Flasks were incubated for 1 h and two sub-samples (2 ml) of liquid were removed to sterile tubes, and centrifuged at 10,000 rpm at 4°C for 20 min. The supernatant was decanted and stored at −70°C until further use. Bioethanol estimation (mg^-1 ^wheat straw/bran) was performed using the QuantiChrom™ Bioethanol Assay Kit (DIET-500) (BioAssay Systems, CA, USA) according to manufacturer's instruction. Results were based on duplicate analyses conducted for each sub-sample.

### Estimation of acetic acid and xylitol

Acetic acid and xylitol content of the culture supernatants were determined enzymatically using the Megazyme™ acetic acid and Xylitol assay kits (Megazyme, Co. Wicklow, Ireland) according to manufacturer's instruction. Results were based on duplicate analyses conducted for each sub-sample.

### Estimation of fungal biomass

Fungal biomass was estimated based on the glucosamine content of cell wall chitin. Chitin was hydrolysed into *N*-acetyl glucosamine as previously described by Scotti et al. (2001), which was assayed by the modified colorimetric method described by Ride and Drysdale (1972).

### Enzyme activity

For enzyme assays, SSC was conducted using the medium described by Mishra et al. (1984) (pH 7). Fungal cultures contained 91% initial moisture and were incubated at 30°C. For cellulase assays, flasks were harvested after 4 days of aerobic growth because an initial screen identified this as the time point when cellulase activity reached maximal levels for all the *F. oxysporum *strains (data not shown). Following SSC, cultures were supplemented with 10 ml of distilled water and were incubated at 25°C, 150 rpm for 1 h before being transferred to sterile tubes and centrifuged at 10,000 rpm for 20 min. Supernatants were harvested, flash-frozen in liquid nitrogen and stored at −70°C until further use. Total protein content in the supernatant was determined using the Bradford assay and bovine serum albumin (BSA) as a standard (Bradford 1976). Exoglucanase (EC 3.2.1.91) activity was measured using 1% Avicel (Sigma Chemical, St. Louis, USA) in 100 mM sodium acetate buffer (pH 4.8), as described by (Wood & Bhat 1988) and activity was expressed in nkat μg^-1 ^of crude protein. The activity of β-glucosidase (EC 3.2.1.21), β-xylosidase (EC 3.2.1.37), endoglucanase (EC 3.2.1.4) and endoxylanase (EC 3.2.1.8) in the supernatant was determined as described by Thygesen et al. (2003) and was expressed in nkat μg^-1 ^of crude protein. In each enzyme assay, the specific activity of two commercially available cellulase enzyme mixes, Celluclast^® ^from *Trichoderma reesei *(Cat. no. 9012-54-8, Sigma Chemical, St. Louis, USA) and Novozyme 188 from *Aspergillus niger *(Cat. no. C6105, Sigma Chemical, St. Louis, USA) were also estimated, as was that of a mixture of Celluclast^® ^(83% v v^-1^) and Novozyme 188 (17% vv^-1^), as described earlier by Thygesen et al. (2003).

When assaying alcohol dehydrogenase (ADH) (EC 1.1.1.1) activity, SSC was conducted as described for cellulase enzyme assays, except that incubation conditions were 4 days aerobic followed by 4 days oxygen-limited growth. Following SSC, solid residue (including fungal mycelium) was washed twice with sterile distilled water. Samples were flash-frozen in liquid nitrogen and stored at −70°C until use. Cell extract was obtained as described by Kayali et al. (2005). The total protein in the extract was determined as above and ADH activity was assayed and expressed in nkat μg^-1 ^of protein in the cell free extract as described by Ke et al. (1995). Commercially available ADH from *Saccharomyces cerevisiae *(Cat. No. A3263, Sigma Chemical, St. Louis, USA) was also included in these assays.

### Statistical analysis

See supplementary information for information regarding data distribution, transformation and pooling. The significance of treatment effects was analysed within the Statistical Package for the Social Sciences (SPSS 11.0, SPSS Inc.) by either (i) normally distributed data - one-way ANOVA with Post Hoc pair wise Least Significance Difference (LSD) comparisons (P = 0.050), or (ii) non-normally-distributed data - the Kruskal-Wallis H test. Correlation analysis between specific enzyme activities and either bioethanol yield or fungal biomass data was performed by one-tailed correlation analysis conducted using mean data values (non-normal data: Spearman Rank; normal data: Pearson product moment) within SPSS. Correlation analysis between bioethanol yield and fungal biomass data (transformed) was performed using one-tailed Pearson product moment correlation analyses within SPSS.

## Results

### Collection and identification of fungi

**Figure 1:**
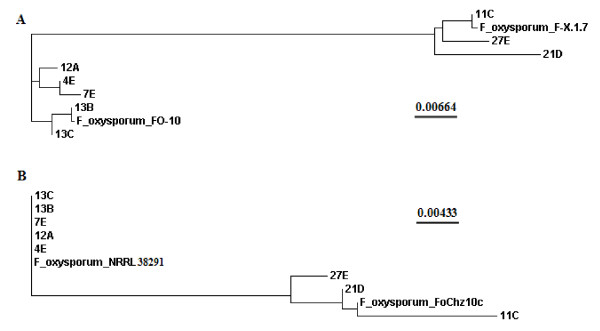
**Genetic diversity among eight strains of *Fusarium oxysporum*, based on DNA sequence data from fragments of the (A) internal transcribed spacer (ITS) region of nrDNA (B) elongation factor-1 alpha (*EF-1α*) gene**. Sequences analysed were derivedfrom *F. oxysporum *strains 4E, 7E, 12A, 13C, 11C, 13B, 21D, 27E or *F. oxysporum *sequences within the NCBI Nucleotide database (http://www.ncbi.nlm.nih.gov/nuccore): F-X.1.7-030520-03 (GenBank No. EU364857.1), FO-10 (GenBank No. AY928417.1), FoChz10c (GenBank No. EU313533.1) and NRRL 38291 (GenBank No. FJ985370.1). The ITS was amplified using PCR primers ITS4 and ITS5 as described by White et al. (White et al. 1990). The 5' portion of *EF-1α *was amplified using the primers EF-1 and EF-2 as described by O'Donnell et al. (1998). DNA sequences were aligned using European Bioinformatics Institutes's ClustalW2 tool (www.ebi.ac.uk) (Larkin et al. 2007) and phylogenetic trees were generated using the Neighbor-joining method (Saitou & Nei 1987)

Using a selective medium, 17 strains of *F. oxysporum *strains were isolated from agricultural soil, peat and plant samples collected from different sites in Ireland (Additional file 1: Table S1). These single-spored isolates were identified based on conidial morphology and species-specific PCR analyses (results not shown). Six strains (4E, 7E, 11C, 12A, 13C and 27E) (see supplementary information for IMI accession number) were selected from the original population, based on preliminary studies (data not shown) focussed on optimising the growth and incubation conditions for straw/bran bioconversion to bioethanol in solid-state culture. These six strains were genetically distinct, as determined by comparative analysis of DNA sequence; as expected (Tanabe et al. 2004) the genetic diversity was greater within ITS as compared to *EF-1*α sequences (Figure 1A &1B).

### Determination of the optimal growth and culture conditions for biomass and bioethanol production from a non-alkali-treated straw/bran mix

The effect of growth media and incubation conditions on the ability of *F. oxysporum *strains 4E, 7E, 11C, 12A, 13C and 27E to both colonise and release bioethanol from non-alkali-treated straw/bran was studied here. Although the optimum conditions were variable depending upon the strain, the temporal conditions that were most favourable for highest bioethanol release were 4 days of aerobic followed by 4 days of oxygen-limited growth (Additional file 1: Figure S1). Of the six different minimal media tested, the media described by Mishra et al. (1984) was the most favourable for bioethanol production for all six strains: the media also supported relatively high fungal growth (Additional file 1: Figure S2). On the basis of temperature and pH experiments conducted in this study, it was observed that bioethanol production by *F. oxysporum *was higher at temperatures at or above 25°C, peaking at 35°C and a pH of 7 for most of the strains (Additional file 1: Figures S3 and S4). It was observed that bioethanol yield generally increased with increasing initial moisture content, but *F. oxysporum *biomass levels were not significantly different at 80 vs. 91% (vw^-1^) (P > 0.05) (Additional file 1: Figure S5). In the SSC experiments, *F. oxysporum *strain 27E consistently produced more biomass than the five other strains tested, but it did not give the highest bioethanol yields (Additional file 1: Figures S1 - S5).

### Inter-strain variation in biomass and bioethanol yields from non-alkali-treated straw/bran

**Figure 2:**
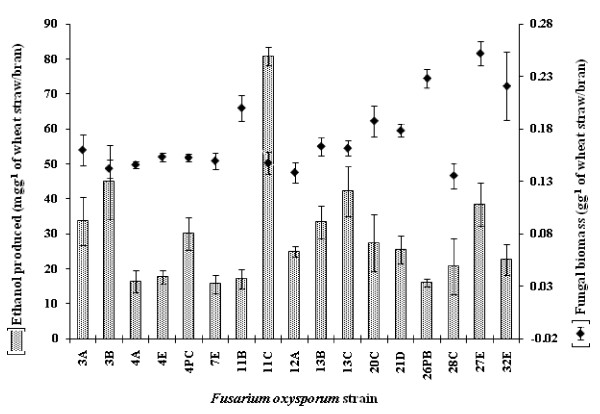
**Growth and ethanol production by strains of *Fusarium oxysporum *cultivated on a straw/bran lignocellulosic substrate (10:1 ratio of straw to bran)**. Fungal isolates were grown in solid-state culture on minimal medium, pH 7 (Mishra et al. 1984) supplemented with 1 g milled straw/bran (initial moisture content was 91% vw^-1^). Cultures were incubated at 35°C for 4 days of aerobic followed by 4 days of oxygen-limited conditions. Ethanol produced in the culture was estimated using QuantiChrom^™ ^Ethanol Assay Kit (DIET-500) (BioAssay Systems, USA) according to manufacturer's instruction. Fungal biomass was estimated as described earlier by Scotti et al. (Scotti et al. 2001). Bars indicate SEM (LSD _0.05 _bioethanol = 8.93; LSD _0.05 _biomass = 0.023).

Based on the above experiments conducted using six fungal strains, the optimal conditions for bioethanol production from the unhydrolysed straw/bran mix in solid state culture were growth in the minimal medium described by Mishra et al. (1984), initial moisture content of 91% (vw^-1^), pH7 and incubation at 35°C for 4 days under aerobic followed by 4 days under oxygen-limited conditions. Using these conditions, a comparative study of 17 *F. oxysporum *strains with respect to their ability to colonise and release bioethanol from straw was completed (Figure 2). The bioethanol yield varied significantly according to the strain used (P ≥ 0.050). *F. oxysporum *strain 11 C released the highest levels of bioethanol g^-1 ^of wheat straw/bran (up to 80 mg). At the other extreme, strains 4A and 7E produced only 16 and 15 mg of ethanol g^-1 ^of wheat straw/bran, respectively, under optimised conditions (Figure 2).

There was no correlation between bioethanol produced by the strains of *F. oxysporum *and biomass levels (*r *≤ 0.323; *n *= 36; *P *≥ 0.050) (Figure 2). Strains 32E, 27E, 26 PB and 11B produced significantly higher biomass, as compared to 11 C (≥ 0.20 vs. 0.14 gg^-1 ^of wheat straw/bran; *P *< 0.050) (Figure 2). However, the bioethanol production by these three strains was significantly lower compared to 11C (≤ 38 vs. 80 mgg^-1 ^of wheat straw/bran); (*P *< 0.050) (Figure 2).

Experiments were then conducted to determine the amount of bioethanol these fungi could release from alkali-treated straw under the culture conditions described by Christakopoulos et al. (1991a). After 6 days of oxygen-limited growth, mean bioethanol yields from alkali-treated straw were 221 and 326 mgg^-1 ^for *F. oxysporum *strains 7E and 11C, respectively. This corresponds to 54.5 and 80.2% of the theoretical yield, based on previous calculations (Christakopoulos et al. 1991a).

### Inter-strain variation in acetic acid and xylitol yields from non-alkali-treated straw/bran

**Figure 3:**
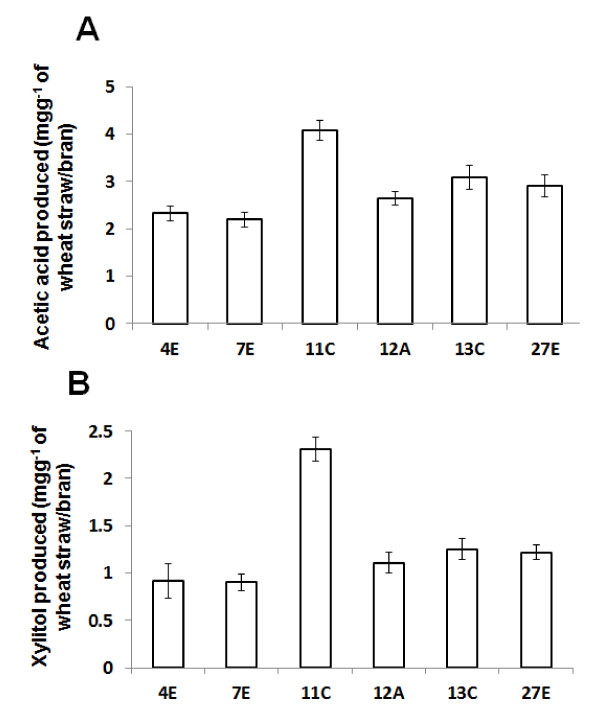
**Acetic acid (A) and xylitol (B) produced as a by-product during bioethanol production by different strains of *Fusarium oxysporum *cultivated on a straw/bran lignocellulosic substrate (10:1 ratio of straw to bran)**. Fungal isolates were grown in solid-state culture on 1 g straw/bran supplemented with 5 ml^-1 ^minimal medium, pH 7 (Mishra et al. 1984) (initial moisture content was 91% vw^-1^). Cultures were incubated at 35°C for 4 days of aerobic followed by 4 days of oxygen-limited conditions. Acetic acid and xylitol content of the culture supernatant were determined enzymatically using the Megazyme^™ ^acetic acid and Xylitol assay kit respectively (Megazyme, Ireland) according to manufacturer's instruction. Bars indicate SEM (LSD_0.05 _acetic acid = 0.45478; LSD_0.05 _xylitol = 0.3004)

The levels of acetic acid and xylitol produced during the bioconversion of non-alkali-treated straw/bran to bioethanol were determined for six strains of *F. oxysporum *(Figure 3). Similar to bioethanol, acetic acid and xylitol levels varied significantly according to the strain used (*P *< 0.050). *F. oxysporum *strain 11C produced the highest levels of both acetic acid and xylitol g^-1 ^of wheat straw/bran (4.07 and 2.3 mg, respectively) (Figure 3). There was a significant correlation between the bioethanol yield and either the acetic acid or xylitol yielded by the six *F. oxysporum *strains (*r *> 0.80214; n = 36; *P *< 0.050).

### Cellulase, xylanase and alcohol dehydrogenase activity during growth on non-alkali-treated straw/bran

**Figure 4:**
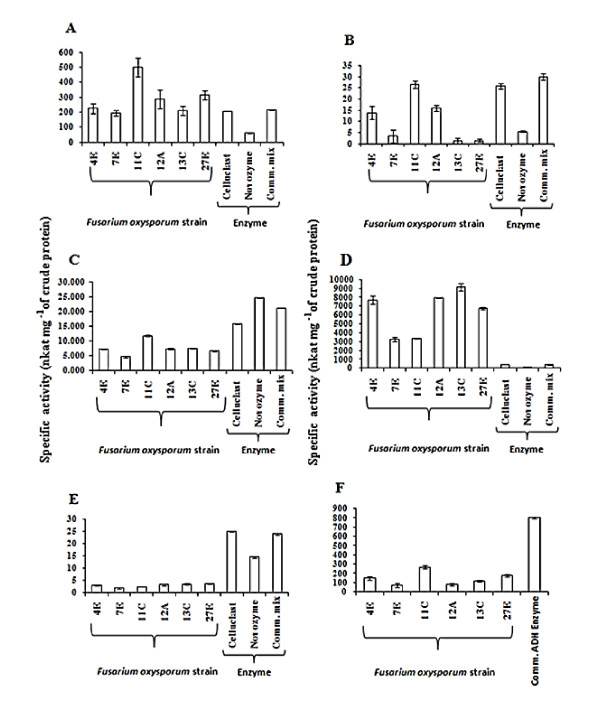
**Specific activity of the major cellulases, xylanase and alcohol dehydrogenase produced by strains of *Fusarium oxysporum *during growth on wheat straw/bran**. Specific activity of (**A**) endoglucanase (EC 3.2.1.4), (**B**) exoglucanase (EC 3.2.1.91), (**C**) β-glucosidase (EC 3.2.1.21), (**D**) endoxylanase (EC 3.2.1.8), (**E) **β-xylosidase (EC 3.2.1.37) and (**F**) alcohol dehydrogenase (ADH) (EC 1.1.1.1) as determined for *F. oxysporum* strains 11C, 12A, 13C, 27E, 4E and 7E and commercial enzyme preparations, i.e. Celluclast^®^, Novozyme 188, Commercial mix. (83% vv^-1 ^Celluclast^®^, 17% vv^-1 ^Novozyme 188) and ADH (Sigma Chemical, St. Louis, USA). Fungal isolates were grown in solid-state culture on minimal medium, pH 7 (Mishra et al. 1984) supplemented with 1 g milled straw/bran (initial moisture content was 91% vw^-1^). Cultures were incubated at 30°C. Activities of cellulases enzymes in the water extract of the fermented straw (after 4 days of aerobic growth) were determined as described earlier by Wood & Bhat (1986) and Thygesen et al. (Thygesen et al. 2003). ADH activity in the cellular extract of the fermented straw (after 4 days of aerobic and 4 days oxygen-limited growth) were determined as described earlier by Kayali et al. (Kayali et al. 2005) and Ke et al. (*Ke et al*. 1995). Total protein in the extracts were measured by Bradford assay (Bradford 1976) and specific activity of the enzymes expressed as nkat μg^-1^of crude protein. Bars indicate SEM (LSD _0.05 _for parts A - F = 89.92, 6.85, 0.52, 962.96, 0.48 and 60.41, respectively)

The activity of the major cellulase and xylanase enzymes during growth on straw/bran mix was investigated for six *F. oxysporum *strains that, as shown in the SSC optimisation studies (Additional file 1: Figures S1 - S5), differed in their ability to colonise and release bioethanol from this lignocellulosic material. The data shows there was inter-strain variability in the specific activity of the major cellulases and xylanase enzymes (Figure 4A to 4E), but there was no correlation between bioethanol levels produced by the strains and the activity of five major enzymes (*r *≤ 0.657; n = 6; *P *≥ 0.050).. The specific activity of endoglucanase secreted by most of the six *F. oxysporum *strains was significantly higher than that of two commercial cellulases enzyme mixes (*P *< 0.050) (Figure 4A), and endoxylanase activity of strain 11C, 12A and 4E was significantly higher than that of a commercial enzyme from A. *niger (P *< 0.050) (Figure 4B). The specific activity of endoxylanase secreted by the strains 12A, 13C and 27E was 18 to 22 times higher than that of the commercial enzyme preparations (*P *< 0.050) (Figure 4D). Overall, strains 11C, 12A and 4E had higher specific cellulase and xylanase activities, as compared to the other three strains (Figure 4A to 4E), and activity was at a high enough level for commercial exploitation. No association was recorded in terms of alcohol dehydrogenase, there was no correlation between bioethanol produced and alcohol dehydrogenase (ADH) activity (*r *≤ 0.257; n = 36; *P *≥ 0.050) for the studied strains. While, inter-strain variation did exist with strain 11C exhibiting the highest enzyme activity, the ADH levels were 3 fold lower than that of the commercial *S. cerevisiae *enzyme tested (*P *< 0.050) (Figure 4F).

## Discussion

One of the major factors affecting the ability of *F. oxysporum *to produce bioethanol from straw is the degree of straw delignification (Christakopoulos et al. 1991a). It should be noted that this study differs from many others in that the focus was on the ability of strains of the fungus *F. oxysporum *to release bioethanol from unprocessed wheat straw. The performance of filamentous fungi in lignocellulosic bioconversion is affected by various culture conditions (Singh et al. 1992) and thus we set out to determine the optimal conditions for *F. oxysporum*-mediated bioconversion of non-alkali-treated straw to bioethanol. As reported earlier by Christakopoulos et al. (1991a), a combination of aerobic and non-aerobic growth phases is necessary for the *F. oxysporum*-mediated biodegradation of straw and fermentation of the resulting sugars to bioethanol. Previous studies suggested that the source of nitrogen in the minimal media plays an important role in the both the production of cellulase by *Clostridium thermocellum *(Garcia-Martinez et al. 1980) and anaerobic growth and product formation by *S. cerevisiae *(Albers et al. 1996). Thus the effect of medium on bioethanol yields observed in this study may have been due to different nitrogen sources.

Experiments conducted by Christakopoulos et al. (1989) indicated that the optimum temperature and pH for the direct conversion of cellulose to ethanol by *F. oxysporum *is 34°C and 5.5 - 6, respectively. *F. oxysporum *can grow at a temperature up to 40°C in solid media and up to 45°C in liquid media (Linfield 1986). Of course, the optimal temperature and pH for growth may not be coincident with those for enzyme activity, as is the case with *T. reesei *(Bailey et al. 1993; Tabka et al. 2006). Because microbial growth, enzymatic hydrolysis and the fermentation phases are carried out synchronically in CBP, it is very important to find culture conditions that are optimal for all these processes. Rapid fungal growth is not necessarily a desirable characteristic for CBP as it may divert sugars towards biomass rather than bioethanol production. It may be that inter-strain variability in mycelial structure rather than biomass is a major determinant of substrate hydrolysis capacity; Domingues et al. (2000) showed that the mycelial structure of *T. reesei *was a major factor influencing cellulase production and substrate hydrolysis. It would be interesting to compare the mycelial structure of the strains used in this study, particularly those that differed significantly in cellulase activity on straw/bran.

The maximum bioethanol yield obtained under the optimised SSC conditions from untreated wheat straw/bran was far below industrially exploitable yields. But it was higher than the levels recently reported for the fungi *N. crassa, Phanerochaete chrysosporium, Gloeophyllum trabeum and T. reesei *(Dogaris et al. 2009; Rasmussen et al. 2010; Shrestha et al. 2010). More importantly this is the highest reported bioethanol yield from any unprocessed lignocellulosic material. When using alkali-treated straw, Christakopoulos et al. (1991a) observed that *F. oxysporum *strain F3 produced up to 275 mg bioethanol g^-1 ^of substrate, corresponding to 67.8% of the theoretical yield. Using similar culture conditions and alkali-treated straw, we found that *F. oxysporum *strain 11C could yield up to 80.2% of the theoretical yield of bioethanol. This yield was also higher than that reported for other fungi grown on pre-treated agricultural waste (Deshpande et al. 1986; Mizuno et al. 2009; Karimi et al. 2006; Okamoto et al. 2011; Goshadrou et al. 2011). When unprocessed straw was used, strain 11C produced 5.3-fold more bioethanol than strain 7E. But in case of alkali-treated straw this difference was 1.5-fold. This indicates that strain 11C is more efficient than strain 7E in converting both delignified and untreated straw to bioethanol, but the difference was more acute in the latter case. Chemical de-lignification represents a significant cost in lignocellulose bioprocessing and the development or identification of factors that could contribute to alternative approaches could reduce the cost of bioethanol production, particularly if such a factor were a component part of a CBP process.

As stated in the introduction, acetic acid and xylitol are undesirable by-products of lignocellulose fermentation by *F. oxysporum *produced as a result of redox imbalance and this diverstion of xylose substrate reduces bioethanol yield (Panagiotou et al. 2005a; Panagiotou et al. 2005b; Panagiotou et al. 2005c). Compared to *F. oxysporum *strain F3 which released 0.2 g of acetic acid g^-1 ^of cellulose (Panagiotou et al. 2005a), the strains used in the present study released significantly less acetic acid which may be due to the slow release of glucose from the untreated straw. The correlation between bioethanol and xylitol production indicate that strains which are more efficient in releasing xylose also produce more xylitol along with bioethanol. Variations in both substrate and growth conditions mean that a direct comparison is not possible with enzyme activities previously reported for *F. oxysporum*, but Panagiotou et al. (2003) also observed higher activity of endoglucanase and endoxylanase enzymes compared to exoglucanase, β-glucosidase and β-xylosidase in the culture supernatant of *F. oxysporum *strain F3. Inter-strain variation in ADH activity is quite common in brewer's yeast (Radler & Schutz 1982; Ca et al. 1996). There are reports of inter-species but not of inter-strain variation in ADH activity in *Fusarium *(Kayali et al. 2005). While going through the *F. oxysporum *f. sp. *lycopersici *(strain 4,287) genome which was sequenced and annotated by the Broad Institute (http://www.broadinstitute.org/) it was observed that it encodes seven genetically-distinct multicopy *ADH *gene variants. Such variation most likely contributes to inter-species variation in bioethanol production capacity and it would be of interest to compare the transcription of these genes at the strain and inter-strain level during SSC.

In conclusion, *F. oxysporum *strain 11C yielded impressive amounts of bioethanol from delignified wheat straw under laboratory condition and even has the potential to be employed as a CBP agent for untreated straw or other lignocellulosic materials. This study has also generated a quick and large scale screening process that could be used for screening microorganisms to check their suitability as CBP agents. In addition to culture conditions, degree of colonisation and enzyme activities, other determinants that likely contribute to strain-dependent differences in bioethanol production are the capacity for cellular intake of the hexose and pentose sugars, ability to ferment pentose sugars to bioethanol, the tolerance of the strain towards bioethanol and accumulation of undesirable by-products like acetic acid and xylitol. These determinants are being further investigated in our laboratory. On the basis of the results presented here, we discriminated which strains of *F. oxysporum *to use in a comparative transcriptomics study ongoing in our laboratory, the aim of which is to identify candidate genes that contribute to the relative higher bioethanol production capacity of strain 11C.

## Competing interests

The authors declare that they have no competing interests.

## Supplementary Material

Additional file 1 **Supplementary information**. Click here for file
