# Supplementary material for: Exploiting the inter-strain divergence of Fusarium oxysporum for microbial bioprocessing of lignocellulose to bioethanol
Source: AMB Express. 2012 Mar 15;2:16. doi: 10.1186/2191-0855-2-16 (PMC3366892; doi:10.1186/2191-0855-2-16)
Supplement: Additional file 1 — Supplementary information. [file 2191-0855-2-16-S1.DOC]

Supplementary information

Exploiting the evolutionary divergence of *Fusarium oxysporum* for microbial bioprocessing of lignocellulose to bioethanol

Ali, Shahin S.^1a^; Khan, Mojibur^1a^; Fagan, Brian^1^; Mullins, Ewen^2^ and Doohan, Fiona^1^**^*^**

^1^Molecular Plant-Microbe Interactions Laboratory, School of Biology and Environmental Science, University College Dublin, Dublin 4, Ireland.

^2^Dept. of Crop Science, Teagasc Crop research Centre, Oak Park, Carlow, Ireland.

^a^Joint first authors

**^*^**Corresponding author:

Email: fiona.doohan@ucd.ie

Fax: 0035317161102

Phone: 0035317162248

**Supplementary Materials & Methods**

**Isolation of *Fusarium oxysporum***

1g of soil was suspended in 5ml of sterile H_2_O and this was incubated at room temperature at 150 rpm for 1h. Thereafter, 100µl aliquots of the supernatant were spread upon Komada’s selective medium. The plates were incubated at 25ºC under dark and emerging colonies were transferred to potato dextrose agar (PDA; Oxoid Ltd, UK) plates. PDA plates were incubated at 25ºC and, after 3-5 days, mycelium from the leading colony edge was used to inoculate a fresh PDA plate. These plates were incubated at 25ºC and after 5 days fungi were again sub-cultured again onto a fresh PDA plate and allowed to sporulate under light. A single spore was harvested using a sterile needle and was inoculated onto PDA. PDA cultures were grown at 25ºC for 5 days and resulting mycelium was submersed in 30% glycerol (vv^-1^), flash-frozen in liquid nitrogen and was stored at -70ºC. Putative *Fusarium* isolates were differentiated from other isolates of closely related species based on conidial morphology as described earlier by Burgess et al. (1989). Some of the important strains (with IMI accession number) were also deposited to CABI’s Microbial Services under the United Kingdom National Culture Collection (UKNCC) for open access. Prior to use, fungal isolates were sub-cultured onto PDA plates and incubated at 25ºC for 5 days.

**Statistical analysis**

The following data sets were normally distributed as determined using the Ryan Joiner test (Ryan and Joiner, 1976) within Minitab (Minitab release 13.32©, 2000 Minitab Inc.): bioethanol and biomass data from the temporal SSC studies, the biomass data from SSC minimal medium experiments, and exoglucanase and alcohol dehydrogenase activity data. All other data sets were non-normally distributed. Most of these non-normally distributed data were transformed to fit a normal distribution using the Johnson transformation (Johnson, 1978) within Minitab (Minitab release 13.32©, 2000 Minitab Inc.). The homogeneity of data sets across replicate experiments was confirmed by one-tailed correlation analyses conducted using mean data values (non-normal data: Spearman Rank; normal data: Pearson product moment) conducted within the Statistical Package for the Social Sciences (SPSS 11.0, SPSS Inc.) (*r* ≥ 0.610; *P <* 0.050) (Snedecor and Cochran, 1980). Therefore, data sets from the replicate experiments were pooled for the purposes of further statistical analysis.

**References:**

Burgess, L.W., Nelson, P.E., Toussoun, T.A., 1989. Stability of Morphological Characters of *Fusarium nygamai*. Mycologia., 81, 480-482.

Johnson, N.J., 1978. Modified t tests and confidence intervals for asymmetrical populations. *J. Am. Stat. Assoc.*, 73, 536-544.

Ryan, T.A., Joiner, B.L., 1976. Normal probability plots and tests for normality. Minitab Statistical Software: Technical Reports. The Pennsylvania State University, State College, PA. Available from MINITAB, Inc.

Snedecor, G.W., Cochran, W.G., 1980 Statistical Methods. The Iowa State University.

**Table S1:** Code and origin of *Fusarium oxysporum* strains.

| Code | Sample | Origin | IMI accession number |
| --- | --- | --- | --- |
| 3A | Peat | Gortahork, Co. Donegal, Ireland | - |
| 3B | Oat rhizosphere soil | Manorcunningham, Co. Donegal, Ireland | - |
| 4A | Grass rhizosphere soil | Omagh, Co. Tyrone, Northern Ireland | - |
| 4E | Grass rhizosphere soil | Pontzpass, Co. Tyrone, Northern Ireland | - |
| 4PC | Grass rhizosphere soil | Pontzpass, Co. Tyrone, Northern Ireland | - |
| 7E | Potato rhizosphere soil | Raphoe, Co. Donegal, Ireland | 501116 |
| 11B | Potato rhizosphere soil | Bulked sample from various Irish locations | - |
| 11C | Potato rhizosphere soil | Bulked sample from various Irish locations | 501118 |
| 12A | Potato rhizosphere soil | Bulked sample from various Irish locations | - |
| 13B | Potato rhizosphere soil | Bulked sample from various Irish locations | - |
| 13C | Potato rhizosphere soil | Bulked sample from various Irish locations | - |
| 20C | Barley rhizosphere soil | Oak Park, Co. Carlow, Ireland | - |
| 21D | Barley rhizosphere soil | Oak Park, Co. Carlow, Ireland | - |
| 26PB | Wheat rhizosphere soil | Co. Offaly, Ireland | - |
| 27E | Wheat rhizosphere soil | Co. Wexford, Ireland | 501117 |
| 28C | Wheat rhizosphere soil | Co. Wexford, Ireland | - |
| 32E | Barley rhizosphere soil | Backweston, Co. Kildare, Ireland | - |


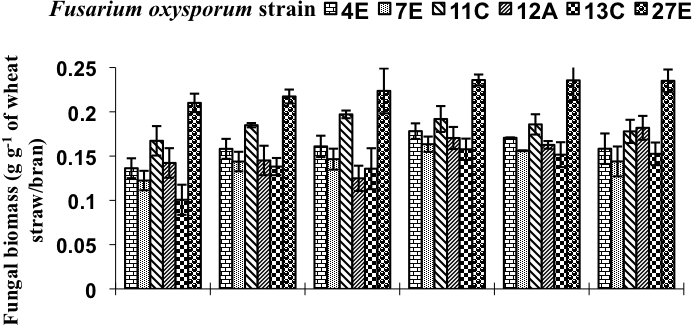


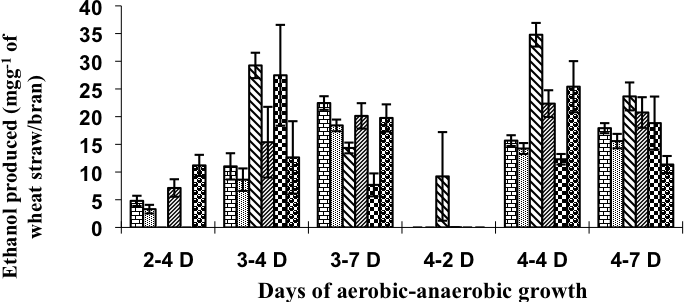


**Fig. S1** Optimisation of lignocellulose culture conditions for *Fusarium oxysporum* strains with respect to duration of aerobic and oxygen-limited growth phases. Wheat straw/bran (10:1 ratio; 1g) was mixed with minimal media (Mishra et al*.* 1984), autoclaved (121^0^C for 15min) in a 100ml Erlenmeyer flask and inoculated with 4ml of fungal conidia (10^6^ condia ml^-1^ of growth medium). Ethanol produced in the culture was estimated using QuantiChrom^TM^ Ethanol Assay Kit (DIET-500) (BioAssay Systems, CA, USA) according to manufacturer’s instruction. Fungal biomass was estimated as described earlier by Scotti et al. (2001). Bars indicate SEM (LSD _0.05_ biomass=0.041, ethanol=6.090).


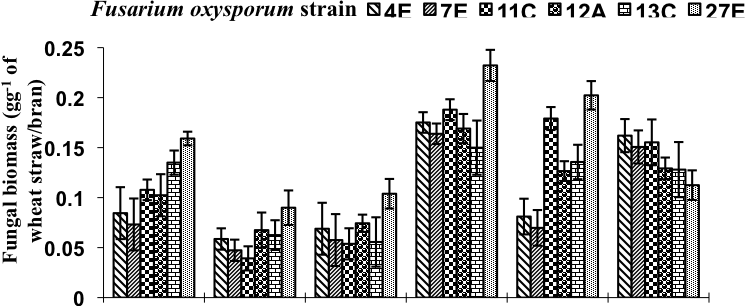


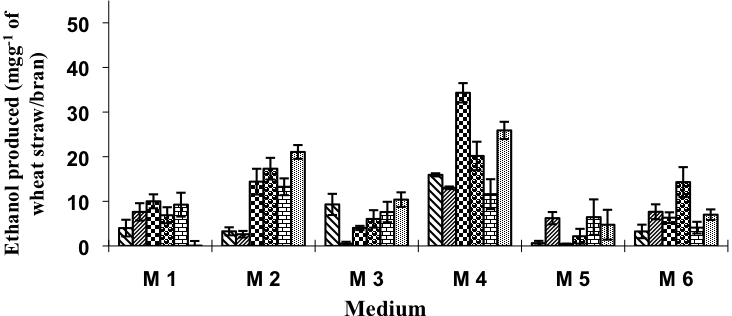


**Fig. S2** Optimisation of lignocellulose culture conditions for *Fusarium oxysporum* strains with respect to minimal medium used. Wheat straw/bran (10:1 ratio; 1g) was mixed with minimal media (see below), autoclaved (121^0^C for 15min) in a 100 ml Erlenmeyer flask and inoculated with 4 ml of fungal conidia (3 x 10^6^ conidia ml^-1^ of growth medium). Ethanol produced in the culture was estimated using QuantiChrom^TM^ Ethanol Assay Kit (DIET-500) (BioAssay Systems, CA, USA) according to manufacturer’s instruction. Fungal biomass was estimated as described earlier by Scotti et al. (2001). Bars indicate SEM (LSD _0.05_ biomass=0.042, ethanol=2.801). Media: M1, Ucida et al. (2003); M2, Crawford (1987); M3, Christakopoulos et al. (1991); M4, Mishra et al. (1984); M5, Bollok and Reczey (2000) and M6, Panagioton et al. (2003).


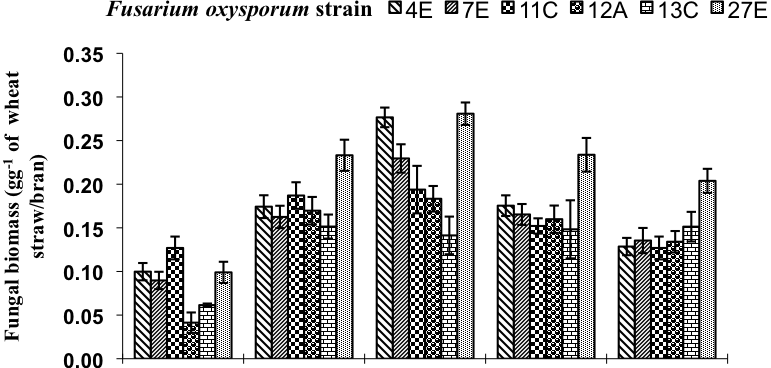


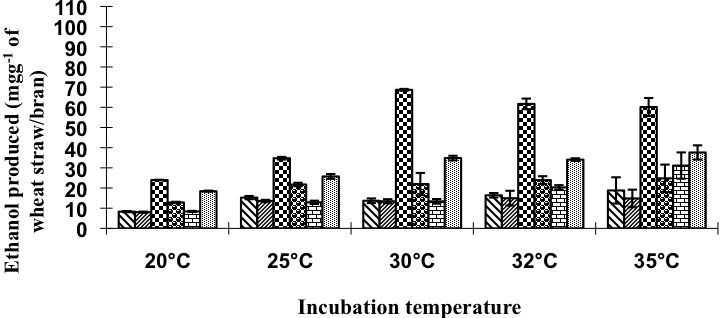


**Fig. S3** Optimisation of lignocellulose culture conditions for *Fusarium oxysporum* strains with respect to incubation temperature used. Wheat straw/bran (10:1 ratio; 1g) was mixed with minimal media (Mishra et al*.* 1984), autoclaved (121^0^C for 15min) in a 100 ml Erlenmeyer flask and inoculated with 4 ml of fungal conidia (3 x 10^6^ conidia ml^-1^ of growth medium). Ethanol produced in the culture was estimated using QuantiChrom^TM^ Ethanol Assay Kit (DIET-500) (BioAssay Systems, CA, USA) according to manufacturer’s instruction. Fungal biomass was estimated as described earlier by Scotti et al. (2001). Bars indicate SEM (LSD _0.05_ biomass=0.046, ethanol=5.409).


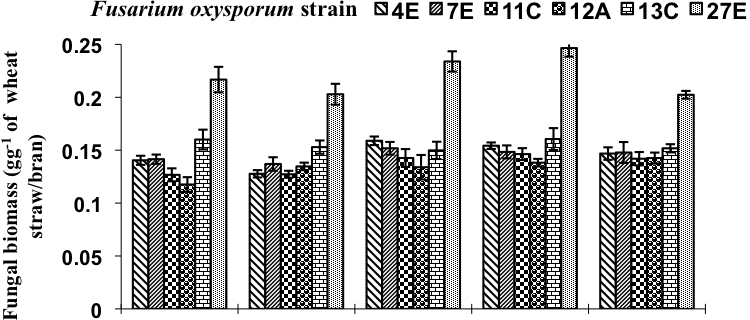


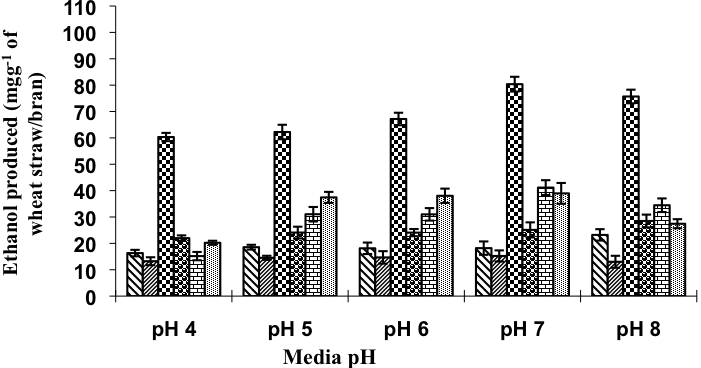


**Fig. S4** Optimisation of lignocellulose culture conditions for *Fusarium oxysporum* strains with respect to the initial pH of the medium. Wheat straw/bran (10:1 ratio; 1g) was mixed with minimal media (Mishra et al*.* 1984), autoclaved (121^0^C for 15min) in a 100 ml Erlenmeyer flask and inoculated with 4 ml of fungal conidia (3 x 10^6^ conidia ml^-1^ of growth medium). Ethanol produced in the culture was estimated using QuantiChrom^TM^ Ethanol Assay Kit (DIET-500) (BioAssay Systems, CA, USA) according to manufacturer’s instruction. Fungal biomass was estimated as described earlier by Scotti et al. (2001). Bars indicate SEM (LSD _0.05_ biomass=0.001, ethanol=13.471).


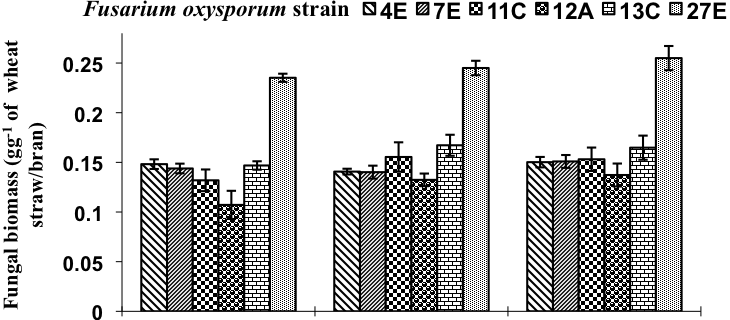


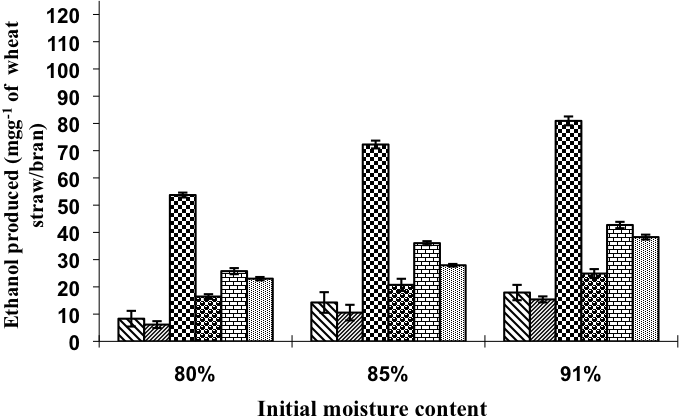


**Fig. S5** Optimisation of lignocellulose culture conditions for *Fusarium oxysporum* strains with respect to the initial moisture content. Wheat straw/bran (10:1 ratio; 1g) was mixed with minimal media (Mishra et al*.* 1984), autoclaved (121^0^C for 15min) in a 100 ml Erlenmeyer flask and inoculated with 4 ml of fungal conidia (3 x 10^6^ conidia ml^-1^ of growth medium). Ethanol produced in the culture was estimated using QuantiChrom^TM^ Ethanol Assay Kit (DIET-500) (BioAssay Systems, CA, USA) according to manufacturer’s instruction. Fungal biomass was estimated as described earlier by Scotti et al. (2001). Bars indicate SEM (LSD _0.05_ biomass=0.023, ethanol=4.925).
